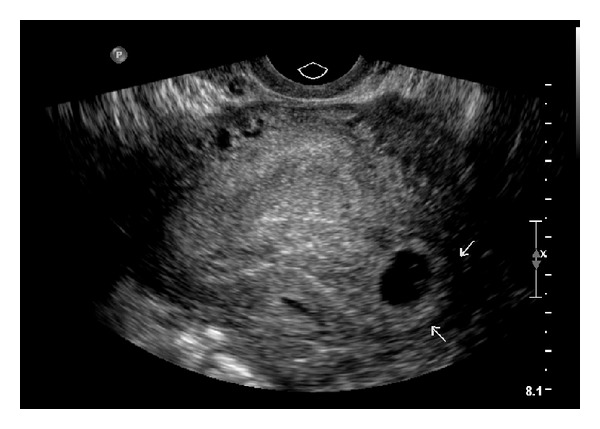# Successful Laparoscopically Assisted Transcervical Suction Evacuation of Interstitial Pregnancy following Failed Methotrexate Injection in a Community Hospital Setting

**DOI:** 10.1155/2014/695293

**Published:** 2014-02-05

**Authors:** Rani B. Fritz, Neal Rosenblum, Kecia Gaither, Alonzo Sherman, Alwyn McCalla

**Affiliations:** ^1^Wayne State University, Detroit, MI 48202, USA; ^2^Brookdale University Hospital and Medical Center, Brooklyn, NY 11212, USA

## Abstract

We report on a case of a patient with an early diagnosed cornual ectopic pregnancy following failed methotrexate treatment. The patient was subsequently taken to the operating room for a laparoscopic guided transcervical suction curettage of the cornual ectopic. The surgery was successful and the patient was followed up until her urine pregnancy test was negative. We conclude that in properly selected patients, cornual ectopic pregnancy may be treated with transcervical suction curettage.

## 1. Introduction

Interstitial pregnancy, commonly referred to as cornual pregnancy, is a rare form of tubal pregnancy accounting for approximately 2–4% of tubal pregnancies [1]. The incidence of interstitial pregnancies is on the rise presumably due to the increase in assisted reproductive technologies [2]. Early diagnosis of interstitial ectopic pregnancies is crucial, as they often remain asymptomatic until rupture occurs and carry a mortality rate as high as 2.5% [3].

Historically, diagnosis of interstitial ectopic pregnancy was most often made intraoperatively in an emergent fashion, following patient presentation with an acute abdomen and/or shock [4]. This would lead to either an interstitial resection or a hysterectomy. With advances in sensitive beta human chorionic gonadotropin (BHCG) assays and ultrasound technologies, earlier diagnosis of interstitial pregnancy is being made. Despite these advances, a large portion of interstitial pregnancies are treated with interstitial resection, even when treated laparoscopically [3]. Removal of the interstitial portion of the uterus carries potential complications in subsequent pregnancies including risks of uterine rupture [5]. Patients with previous interstitial resection will likely undergo elective cesarean section in subsequent pregnancies due to fear of rupture. We report a case of an early diagnosed interstitial pregnancy treated with laparoscopic guided transcervical evacuation following intramuscular methotrexate administration.

## 2. Case Report

The patient was a 37 y/o gravida 7 para 3033 and presented for an ultrasound due to a positive urine pregnancy and vaginal spotting. She stated that her last menstrual period was approximately 7-weeks prior. Aside from vaginal spotting, the patient denied abdominal tenderness or any other complaints. Her past obstetrical history was significant for a full-term normal spontaneous vaginal delivery followed by 2 full-term cesarean sections that were uncomplicated. She had two elective terminations of pregnancy and one first trimester spontaneous abortion. Her gynecological history was significant for a copper intrauterine device (IUD) placed 1 year ago. She denied any history of sexually transmitted diseases. Aside from her cesarean sections and elective terminations, the patient denied any other surgical procedures. Her only medical problem was asthma that was well controlled.

**Figure 1:**
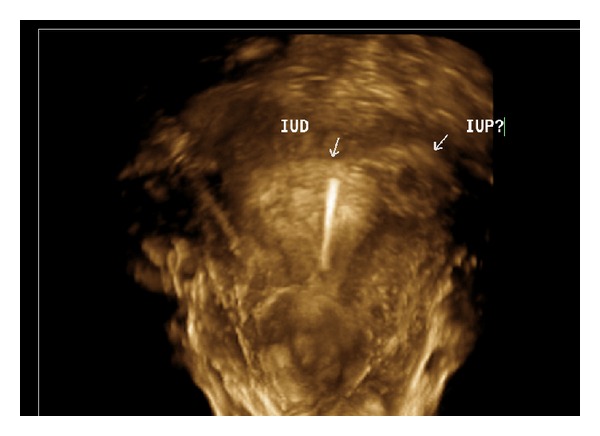


Ultrasound revealed a uterus measuring 9.2 × 9.1 × 6.4 cm, an IUD in the endometrial cavity, normal ovaries bilaterally, no free fluid, and a 1.18 × 1.04 × 1.03 cm gestational sac containing a yolk sac in the left cornual region (Figures 1 and 2). The patient was sent to the emergency department for further evaluation. The patient stated that this was an undesired pregnancy. Upon arrival to the emergency room, her vital signs were stable and her physical exam was unremarkable. Speculum exam did not reveal any evidence of vaginal bleeding and the IUD strings were visualized emanating from the cervical os. At the patient's request, the IUD was removed. Her beta human chorionic gonadotropin (BHCG) value was 8200.0 IU/L and her hemoglobin was 12.5 g/dl.

The patient was counseled regarding her likely diagnosis of an interstitial pregnancy and all treatment options were discussed with her. Following counseling, the patient decided on methotrexate administration. The patient was treated with an intramuscular dose of methotrexate of 50 mg/m^2^. The patient returned asymptomatically for her day 4 BHCG level which revealed 17,023.0 Iu/L. On day 7, her BHCG level revealed 16,389.0 Iu/L with a hemoglobin of 13.1 g/dl. The patient was informed of the inappropriate drop of BHCG and the patient was reassessed in the emergency room. She denied abdominal pains or vaginal bleeding. Her vital signs were stable and her physical exam was benign. A repeated ultrasound was obtained on day 7, which essentially was unchanged from her previous ultrasound. All treatment options were rediscussed with the patient and patient desired surgical management.

A left upper quadrant 5 mm skin incision was made 2 cm below the costal margin in the midclavicular line and a Veress needle was introduced in order to obtain pneumoperitoneum. The veress needle was replaced with a 5 mm trocar and direct intra-abdominal placement was confirmed. Extensive omental and bowel adhesions to the anterior abdominal wall were appreciated. Evaluation of the uterus revealed slight bulging of the left cornual region, approximately 1 cm, lateral to the round ligament without any gross evidence of placental accreta. The remainder of the patient's pelvis revealed otherwise normal uterus, tubes, and ovaries bilaterally. Decision was then made to evacuate the pregnancy via suction curettage. The cervix was dilated using Hegar dilators in order to accommodate a 6 mm suction curette. Under laparoscopic guidance, the curette was gently and easily advanced into the uterine cavity, and with 60 mmHg of pressure, the products of conception were suctioned out with 4 passes. Frozen pathology revealed products of conception. Reevaluation of the left cornual region laparoscopically revealed no evidence of rupture. The patient's skin incision was closed with a 4–0 VICRYL. The patient was sent to the recovery room in stable condition and discharged home the same day.

The patient returned for follow-up three days following her surgery and was asymptomatic, and her BHCG level was noted to be 1470.9 Iu/L. Three weeks later, her urine pregnancy test was negative.

Approximately 3 months following the surgery, the patient had decided to undergo permanent sterilization. She was counseled on all options and decided to undergo hysteroscopic Essure placement. Hysteroscopy was performed which revealed a normal uterine contour and normal ostia bilaterally. The Essure devices were placed bilaterally in standard fashion without difficulty.

## 3. Discussion

The interstitial portion of the fallopian tube originates from the tubal ostium and follows a course away from the uterine cavity to the point at which the isthmic portion of the tube arises from the uterine fundus. This relatively thick portion of tube has a significantly greater capacity to expand before rupture. This may cause a delay in diagnosis and allow the pregnancy to progress further than a tubal ectopic would which may result in catastrophic hemorrhage [3].

It is crucial to diagnose interstitial pregnancies prior to rupture. Along with a high index of suspicion based on risk factors and quantitative serum BHCG levels, ultrasound has been proven to be accurate in the early diagnosis of interstitial ectopic pregnancy. Timor-Tritsch et al. described sonographic findings that are highly specific for the diagnosis of interstitial ectopic pregnancies [6]. Early diagnosis allows for more conservative treatments. These successful treatment modalities have included expectant management [7], systemic methotrexate injection [8], and direct injection of methotrexate into the gestational sac under ultrasound guidance [9]. Various laparoscopic surgical excisional techniques, including cornual wedge resection, cornuostomy, and cornual resections, have also been successfully preformed [3].

All these conservative treatment modalities have their potential advantages and disadvantages. Both intrauterine and tubal pregnancies undergo spontaneous miscarriage and expectant management may be best employed in patients with declining BHCG levels. However, expectant management carries risk of rupture and therefore may be best treated with patient hospitalization, which incurs significant cost and burden on the patient. Systemic methotrexate administration has the advantages of avoiding surgery and maintaining uterine anatomy. The disadvantages include risks inherent to systemic methotrexate injection, necessity for close follow-up and patient compliance, and risks of failure requiring subsequent injections or leading to rupture [10]. Direct injection of methotrexate via ultrasound guidance has been proven successful; however, it may have its limitations. Direct injection requires operator experience and technical expertise and may lead to rupture during the procedure requiring emergent surgery [9]. Laparoscopic cornual excision, although beneficial for the patient because of minimally invasive techniques, still carries risks for uterine rupture for subsequent pregnancies as previously discussed.

In our case, we present removal of an interstitial pregnancy via suction curettage under laparoscopic guidance. Similar, however slightly varied, transcervical evacuation surgeries for interstitial pregnancies have been reported. The first published case was by Sanz and Verosko in 2002, where, although an interstitial pregnancy was successfully treated with multiple doses of methotrexate, a persistent gestational sac was appreciated on ultrasound. Following hysteroscopy, removal of the remainder of the pregnancy with polyp forceps was performed under ultrasound guidance [11]. Three similar successful cases were published in which hysteroscopy was preformed prior to suction curettage and in some other cases following curettage to remove excess placental tissue with hysteroscopic graspers [12–14]. Transcervical resection of cornual ectopic has also been reported leaving behind the placenta and treated with systemic methotrexate [15]. Most similar to our method, Zhang et al. reported successful cases of cornual ectopics treated by laparoscopically guided transcervical suction curettage without use of hysteroscopy [16].

Minelli et al. reported a similar technique in which a hysteroscopically confirmed interstitial ectopic was treated with laparoscopic guided suction curettage, with resection of the remaining gestational debris via Storz resectoscope [17]. Although this method proved successful, using the hysteroscopic resectoscope in cases with interstitial ectopic may incur increased risk of rupture and may adversely disrupt the endometrium for future pregnancies.

In our case, early diagnosis of interstitial pregnancy enabled us to offer our patient more conservative treatments. Methotrexate, a proven successful treatment for interstitial pregnancies, was attempted however unsuccessful. Although it failed, it may have played a role in disrupting the placental implantation, thereby allowing an easier transcervical extraction via suction curettage.

During an interstitial pregnancy, the region may be greatly distended and more prone to rupture. Even minimal manipulation with the suction curette, hysteroscope, or even hysteroscopic medium may rupture the pregnancy. Although using a hysteroscope may be beneficial in that it may allow visualization of the interstitial ectopic, removal of the interstitial pregnancy may be accomplished without the use of a hysteroscope, thereby minimizing uterine/interstitial manipulation and decreasing the chances of inadvertent interstitial rupture. If suction curettage is to be attempted for an interstitial pregnancy, the use of concomitant laparoscopy is crucial so that prompt surgical action may be undertaken if rupture occurs.

The main advantage of this procedure for interstitial ectopic pregnancies is a less invasive approach that preserves uterine anatomy, thereby averting potential compromise to future pregnancies. As compared to medical management, this technique offers quicker resolution. With these advantages stated, proper patient selection is crucial. The cornual ectopic should be nonruptured, the patient should be hemodynamically stable, and the surgical ability to control bleeding should be in place if rupture occurs.

We believe that in properly selected patients combined laparoscopy and suction curettage may be a viable and less invasive treatment option for interstitial pregnancies. Further cases must be trended in order to evaluate its safety and efficacy.

## Figures and Tables

**Figure 2:**